# Smoke exposure and cardio-metabolic profile in youth with type 1 diabetes

**DOI:** 10.1186/s13098-018-0355-0

**Published:** 2018-07-06

**Authors:** Valeria Calcaterra, Jonathan P. Winickoff, Catherine Klersy, Luca Maria Schiano, Rossella Bazzano, Chiara Montalbano, Valeria Musella, Corrado Regalbuto, Daniela Larizza, Hellas Cena

**Affiliations:** 1 Pediatric and Adolescent Unit, Department of Internal Medicine and Therapeutics, University of Pavia, Pavia, Italy; 2 Pediatric Endocrinology Unit, Department of Maternal and Children’s Health, Fondazione IRCCS Policlinico San Matteo, P.Le Golgi n.2, 27100 Pavia, Italy; 3 Department of Pediatrics, Harvard Medical School, MassGeneral Hospital for Children, Boston, MA USA; 4 Biometry & Clinical Epidemiology, Scientific Direction, Fondazione IRCCS Policlinico San Matteo, Pavia, Italy; 5 Department of Public Health, Experimental and Forensic Medicine, Laboratory of Dietetics and Clinical Nutrition, University of Pavia, Pavia, Italy

**Keywords:** Type 1 diabetes, Smoke, Children, Young adults, Cardio-metabolic profile

## Abstract

**Background:**

To evaluate the relationship between smoking and metabolic parameters in patients affected by type 1 diabetes (T1D).

**Patients and methods:**

We enrolled 104 children and young adults (50 females and 54 males) with T1D (aged 16.4 ± 8.6 years). The subjects were divided into three groups according to their smoking habits: no smoking (NS), passive smoking (PS), active smoking (AS). The physical examination of the participants included nutritional status assessment by anthropometry and pubertal stage according to Marshall and Tanner as well as blood pressure measurement. In all patients, metabolic blood assays including fasting blood glucose, insulin, total cholesterol, high-density lipoprotein cholesterol, and triglycerides were measured. Insulin resistance was determined by glucose disposal rate (eGDR). Physical activity was also recorded.

**Results:**

Significant differences in biochemical and functional parameters among the three groups were demonstrated, in particular for systolic (p = 0.002) and diastolic pressure (p = 0.02) and eGDR (p = 0.039). No differences in daily insulin dose (p = 0.75) and glycated hemoglobin (p = 0.39) were observed. AS group had significantly higher blood pressure (p < 0.05) and lower eGDR (p ≤ 0.001) compared to NS and PS. Significant difference was also detected between PS and NS in systolic and diastolic (p = 0.02) pressure and eGDR (p = 0.01). In a multivariable model adjusted for age, gender, BMI and physical activity, smoking habits did not maintain any independent association with metabolic parameters.

**Conclusion:**

This is the first study in a Mediterranean population, looking at tobacco smoke and cardio-metabolic factors in youth with T1D. The relationship between smoking and unfavorable metabolic profile was demonstrated. On the basis of these findings, smoking tobacco should be considered an important modifiable risk factor for young patients with diabetes mellitus, highlighting the need for intensified smoking prevention and cessation programs.

## Background

Type 1 diabetes (T1D) is a chronic and heterogeneous disease caused by autoimmune destruction of pancreatic beta cells, leading to insulin deficiency. T1D is diagnosed more commonly in children and adolescents [1–4]. Ninety percent of children with diabetes have T1D.

The increased risk for cardiovascular disease (CVD) in T1D [5–9] starts in childhood, and is influenced by a variety of interactions between environmental, genetic, and biological factors [8, 9]. Several studies have documented that smoking increases the risk of premature mortality and microvascular/macrovascular complications in adults with diabetes mellitus [10–12]. In adolescents with type 1 diabetes (T1D), active tobacco smoking worsens glycemic control and is associated with a poorer cardiovascular risk profile [13, 14].

It is not entirely clear whether the increased morbidity and mortality in smokers is due to atherogenic metabolic profile or due to the direct toxic effects of nicotine and other toxic substances in cigarettes on the cardiovascular milieu. Indeed, smoking reduces insulin sensitivity and induces insulin resistance enhancing cardiovascular risk factors such as elevated plasma triglycerides, decreased high-density lipoprotein cholesterol and hyperglycemia [15–18]. Several studies show that smoking is associated with metabolic abnormalities and increases the risk of metabolic syndrome (MS) [11, 19, 20].

Few prior studies have evaluated the relationship between active and passive smoking in juvenile type 1 diabetic subjects and metabolic parameters related to increased CV risk.

## Patients and methods

We recruited 104 consecutive male and female youths (50 females and 54 males) with T1DM (aged 16.4 ± 8.6 years) from the Pediatric Diabetology Unit at Fondazione IRCCS Policlinico San Matteo. The patients were enrolled between September 01, 2017 and December 01, 2017.

All patients received insulin-therapy with insulin pump (9/104, 8.6%) or multiple injections (95/104, 91.3%) and using the carbohydrate counting meal planning approach [21].

Assessment of dietary habits, physical activity, tobacco smoking and exposure to secondhand smoke through self-report was achieved by an interviewer-administered questionnaire, modified from Turconi et al. [22] to all the enrolled subjects and their parents [23].

A 24 h recall was used to check compliance with the carbohydrate counting meal planning approach.

Participants were also asked the average number of hours in a typical week they participated in physical activity, subsequently they were categorized as physically inactive (0–2 h/week) or physically active (3–8 h/week).

The participants were then divided into three different groups according to their smoking habits: active smokers (n = 18; aged 22.90 ± 6.58 years), passive smokers (n = 28; aged 10.20 ± 4.28 years) and non-smokers (n = 58; aged 17.36 ± 8.84 years).

In this study, the subjects according to their smoking habits were divided into three groups

- Active smokers group (AS): subjects smoking a single cigarette, even a puff in the past 30 days.
- Passive smokers group (PS): subjects who lived with at least one smoker for at least 1 year prior to the study.
- Non-smokers group (NS): subjects who had never smoked.

The study was performed according to the Declaration of Helsinki and with the approval of the Institutional Review Board. After having received information about the nature of the study, the patient’s parents or tutors gave written consent for their child’s participation.

## Anthropometric and clinical assessment

Physical examination of the patients included anthropometric measurement of weight, height, waist circumference, BMI calculation, pubertal stage according to Marshall and Tanner (Tanner) (prepubertal characteristics corresponding to Tanner stage 1) [24, 25] and blood pressure (BP) evaluation. In all patients, metabolic blood assays included fasting blood glucose, insulin resistance, total cholesterol, high-density lipoprotein cholesterol, triglycerides were measured.

Weight was measured with participants not wearing shoes and in light clothing, standing upright in the center of the scale platform facing the recorder, hands at sides and looking straight ahead.

Standing height was measured using a Harpenden stadiometer with a fixed vertical backboard and an adjustable head piece. The measurement was taken on the child in an upright position, without shoes, with their heels together and toes apart, hands at sides, aligning the head in the Frankfort horizontal plane. The child was instructed to stand as tall as possible, taking a deep breath, and holding this position to capture the result.

Waist circumference was measured to the nearest centimeter with a flexible steel tape measure with participants standing, with crossed arms, placing the hands on opposite shoulders. After gently exhaling, the abdominal waist circumference was measured on the horizontal plane between the lowest portion of the rib cage and the uppermost lateral border of the right ilium.

Body mass index (BMI) was calculated by dividing the patient’s weight in kilograms by the square of the height in meters.

Systolic (SBP) and diastolic (DBP) blood pressure readings were taken twice using a mercury sphygmomanometer, after the participant sat comfortably for 5 min, with an appropriately sized cuff on the right arm, which was slightly flexed at heart level. The second BP measurement was used for the analysis.

Blood samples were drawn in the morning, after an overnight fast. Serum glucose was measured using the hexokinase-G-6-PDH method (Abbott Diagnostics, Rome, Italy). Total cholesterol was determined by enzymatic method (Abbott Diagnostics) and HDL-cholesterol by accelerator selective detergent method (Abbott Diagnostics). Triglyceride concentration was measured by the glycerol phosphatase oxidase method (Abbott Diagnostics).

Insulin resistance was determined by estimated glucose disposal rate (eGDR), calculated as follows:

$$eGDR \, \left( {mg = kg = min} \right) = 21.158 \, + \, \left( { -\,0.09*WC} \right) \, + \, \left( { - \, 3.407*HTN} \right) \, + \, \left( { -\, 0.551*HbA1c} \right)$$

where HTN is the presence of hypertension (0 = no, 1 = yes. Elevated SBP or DBP was defined with values exceeding the 95th percentile for age and sex [26] and WC is waist circumference. The eGDR shows good correlation with IR measured by the euglycemic–hyperinsulinemic clamp and has been validated for the estimation of insulin sensitivity in individuals with T1D [27–29]. For these reasons, we utilized eGDR as marker of insulin resistance/sensitivity.

### Statistical analysis

All analyses were carried out using Stata 15.1 (StataCorp, College Station, TX, USA). We considered a 2-sided *p* value < 0.05 as statistically significant. We report separately for smoking groups the mean and the standard deviation (SD) for continuous variables and the counts for categorical variables. We compared them between smoking groups with the Kruskall Wallis test and the Fisher exact test, respectively. We applied the Bonferroni correction for pairwise post hoc comparisons. We fitted multivariable linear regression models to assess the association of smoking with a series of metabolic parameters related to CV (diastolic and systolic blood pressure, total cholesterol and eGDR, while adjusting for physical activity, BMI and gender. We assessed model fit graphically through a residual vs. fitted plot.

## Results

According to the smoking habits, 58 (55.77%) subjects were included in NS, 28 (26.92%) in PS and 18 (17.31%) in AS group. As detailed in Table 1, PS group was younger than NS and AS groups (p < 0.001) and NS group was younger than AS (p = 0.008). Auxological and pubertal stage were different inter groups according to age (p < 0.001). Groups did not differ by gender (p = 0.27), nor physical activity (PA) (p = 0.63).

**Table 1:** Comparison of demographic, clinical and metabolic parameters among no smokers (NS), passive smokers (PS) and active smokers (AS)

Variable	NS (n = 58)	PS (n = 28)	AS (n = 18)	p overall	Post hoc comparison p-value (Bonferroni correction)		
					NS vs PS	NS vs AS	PS vs AS
Age (years)	17.36 ± 8.84	10.20 ± 4.28	22.90 ± 6.58	< 0.001	< 0.001	0.008	< 0.001
Sex (M/F)	32/26	11/17	11/7	0.27			
Weight (kg)	56.49 ± 20.90	39.25 ± 20.14	65.80 ± 10.49	< 0.001	0.001	0.02	< 0.001
Height (cm)	157.23 ± 20.83	140.51 ± 22.17	169.73 ± 7.93	< 0.001	0.018	< 0.001	< 0.001
BMI (kg/m^2^)	21.90 ± 4.40	18.55 ± 4.03	22.72 ± 2.46	< 0.001	0.001	0.34	< 0.001
Waist circumference (cm)	71.84 ± 12.73	64.50 ± 13.79	79.47 ± 9.55	< 0.001	0.01	0.03	< 0.001
Tanner stages							
0 (Tanner stage 1)	11	13	0	< 0.001	< 0.001	0.02	< 0.001
1 (Tanner stage 2–3)	6	10	2				
2 (Tanner stage 4–5)	41	5	16				
Physically active (n, %)	32 (56.1%)	14 (50%)	8 (44.4%)	0.63			
Insulin dose (U/kg/die)	0.70 ± 0.27	0.65 ± 0.28	0.67 ± 0.19	0.75			
Glucose disposal rate (mg/kg/min)	10.17 ± 1.37	11.03 ± 1.58	9.43 ± 1.10	< 0.001	0.01	0.02	< 0.001
Glycated hemoglobin (%)	8.21 ± 1.33	7.84 ± 1.54	8.31 ± 1.05	0.39			
Total-cholesterol (mg/dl)	167.39 ± 39.72	162.96 ± 26.76	176.67 ± 31.58	0.31			
HDL-cholesterol (mg/dl)	58.40 ± 11.23	61.54 ± 14.48	54.22 ± 12.12	0.24			
Triglycerides (mg/dl)	77.03 ± 51.66	56.71 ± 20.32	79.61 ± 40.90	0.10			
Diastolic pressure (mmHg)	69.57 ± 8.02	65.14 ± 7.62	71.39 ± 8.54	0.02	0.02	0.77	0.02
Systolic pressure (mmHg)	110.83 ± 13.48	103.46 ± 11.64	116.11 ± 9.63	0.002	0.02	0.01	0.001

Data are reported as mean ± SD or counts and compared with the Kruskall Wallis test or the Fisher exact test

Clinical and metabolic features of the three groups are reported in Table 1.

**Fig. 1:**
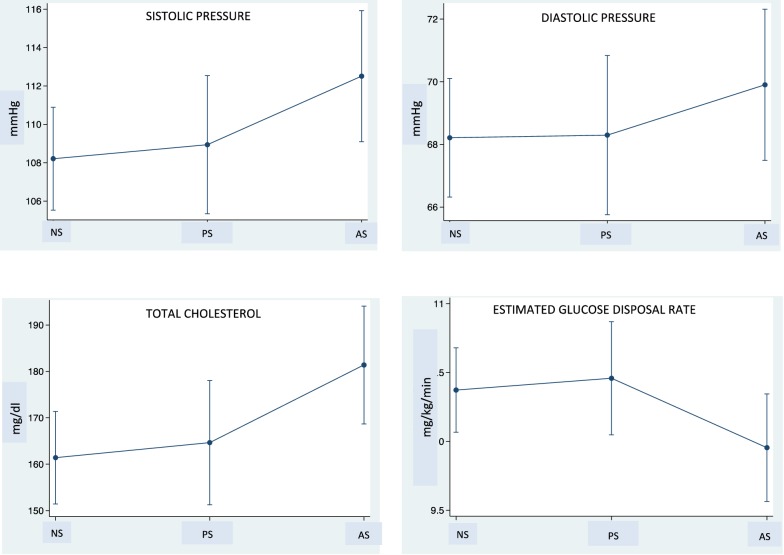
Systolic and diastolic pressure, total cholesterol and estimated glucose disposal rate values in non smokers (NS), passive smokers (PS), active smokers (AS) T1D patients

Adjusted for age, significant differences in biochemical and functional parameters among the three groups were demonstrated, in particular for systolic (p = 0.002) and diastolic pressure (p = 0.02) and eGDR (p = 0.039) (Fig. 1). No differences in daily insulin dose (p = 0.75) and glycated hemoglobin (p = 0.39) were observed.

Noteworthy differences were observed between AS and NS subjects for systolic (p = 0.01) pressure and eGDR level (p = 0.02), between AS and PS subjects for both systolic (p = 0.001) and diastolic (p = 0.02) pressure and eGDR (p < 0.001) and between PS and NS for systolic (p = 0.02) and diastolic (p = 0.02) pressure and eGDR (p = 0.01).

In a multivariable model adjusted for age, gender, BMI and physical activity, smoking habits did not maintain any independent association with metabolic parameters.

## Discussion

This is the first study in a Mediterranean population, looking at tobacco smoke and cardio-metabolic factors in youth with T1D. The data demonstrated that a substantial proportion of youths with type 1 diabetes are active or passive smokers and supports the relationship between smoking and unfavorable metabolic profile.

Globally, over one billion people are regular smokers, and annually an estimated seven million people die as a consequence of smoking [30]. The role of smoking in cardiovascular diseases [31–34] and metabolic abnormalities [11, 19, 20] is well recognized. A dose–response relationship exists with cigarette smoking and the development of metabolic alterations in adults [35].

The mechanisms of smoke induced metabolic and cardiovascular alterations may be partially caused by the toxic effects of nicotine itself. The systemic hemodynamic effects of nicotine are mediated primarily by activation of the sympathetic nervous system. Regarding lipid profile, nicotine induces lipolysis via catecholamine action at β-adrenoreceptors, increasing plasma free fatty acid concentrations, which could result in enhanced synthesis of LDL and lowering of HDL. Additionally nicotine may enhance insulin resistance via increased levels of insulin-antagonistic hormones (catecholamines, cortisol, and growth hormone) and also directly activating AMP-activated protein kinase in adipose tissue via a7 nAChR subtypes mediating the effect of nicotine on insulin sensitivity [17, 18, 36].

Tobacco use is a crucial health problem among young people. Youths around the world take up smoking and use tobacco products at high rates [37]: one out of five adolescents actively smokes tobacco. Besides active smoking, an estimated 40–50% of young children worldwide are regularly exposed to tobacco smoke, primarily by being around smoking parents and/or other household members and two-thirds of all adolescents are exposed to second-hand tobacco smoke [37]. Tobacco smoke exposure has been associated with adiposity, worse neurocognitive development, cognition, and sleep in children, endangering overall health and cognitive functioning demonstrated for the first time, that tobacco smoke is associated in a dose-dependent manner with a four-fold increase risk of metabolic alterations in adolescents [38, 39]. Kalishadi et al. [40] confirmed that both smoking and exposure to smoke are associated with an increased risk of cardiometabolic risk factors and metabolic syndrome in adolescents.

A number of components of the Metabolic Sydrome have been observed in patients with type 1 diabetes and potentially contribute to increased cardiovascular risk. However, few studies have examined the association between smoke and metabolic alterations in youths with diabetes mellitus. Schwab et al. [41] reported that in youth, smokers with type 1 diabetes mellitus, total cholesterol, LDL cholesterol, HbA1c, fructosamine, apolipoprotein B, and serum P-selectin concentrations were higher than non-smokers. Hofer et al. [42], showed a worse cardiovascular risk profile in smokers compared to non-smoking patients, including higher HbA1c, triglyceride, total cholesterol, diastolic blood pressure, and lower HDL. Reynolds et al. [43] confirmed the relationship between smoking, worse lipid profile, and low physical activity levels in youths with type 1 diabetes mellitus. These studies and our own results presage risk of future unfavorable health outcomes in youths with type 1 diabetes mellitus exposed to tobacco smoke.

In our study, difference were also detected between active and passive smoke exposure; active smoking subjects showed higher diastolic and systolic blood pressure values and lower eGDR values than passively exposed subjects.

However, we did not find any significant difference in glucose control in contrast with Gerber et al., Schwab et al., and Hofer et al. [13, 41, 42] who reported worse glucose control and higher HbA1c levels in young T1D smokers compared to non-smokers. Small sample size and a wide age range in our study may explain our inability to find this association.

Since we didn’t have any biologic measures of tobacco smoke exposure [44–48], our study may be vulnerable to misclassification bias, biasing our results to the null hypothesis. In future research we propose to measure cotinine in the urine, saliva, or serum, currently regarded as the best biomarker for exposure of active smokers and non-smokers to environmental tobacco smoke [15, 16].

Moreover sample size may not be sufficient for so many model variables, since the point estimate shows expected directionality but the significance of univariate was not maintained. In the future larger sample size, with a more uniform age distribution, is recommended.

Despite the limitations, studies like ours, focused on specific populations of children suffering from chronic diseases, are very important to raise awareness about the importance of lifestyle interventions, including smoking cessation. Smoke exposure plays a role in the cardio-metabolic profile of youths with T1D, contributing to cardiovascular risk, and supporting the need for intensified smoking prevention and cessation programs for young patients with diabetes mellitus.
